# Attachment and propensity for reporting compassionate opportunities and behavior in everyday life

**DOI:** 10.3389/fpsyg.2024.1409537

**Published:** 2024-07-31

**Authors:** Deanna Varley, Chase S. Sherwell, James N. Kirby

**Affiliations:** ^1^Compassionate Mind Research Group, School of Psychology, University of Queensland, Brisbane, QLD, Australia; ^2^The Matilda Centre for Research in Mental Health and Substance Use, University of Sydney, Sydney, QLD, Australia; ^3^The UQ Learning Lab, School of Education, The University of Queensland, Brisbane, QLD, Australia

**Keywords:** attachment, compassion, experience sampling method, prosocial behavior, ecological momentary assessment

## Abstract

**Introduction:**

Researchers have identified links between anxious and avoidant attachments and difficulties with self-compassion, giving others compassion, and receiving compassion. However, while compassion requires both awareness of opportunities for compassion and compassionate action, little is known about attachment-related differences in reporting compassionate opportunities. Further, most research relies on retrospective-reports that may not accurately assess compassionate behaviors in everyday life.

**Method:**

Consequently, we collected 2,757 experience sampling survey responses from 125 participants (95 women, 27 men, 3 non-binary, *M*_age_ = 18.74, *SD*_age_ = 1.66) to investigate whether attachment anxiety, avoidance, or their interaction were associated with differences in propensity for reporting compassionate opportunities, actions, and emotional responses to opportunities in everyday life across self-compassion, giving compassion, and receiving compassion.

**Results:**

Anxiety was associated with greater likelihood of reporting all types of compassionate opportunities and less positive responses to opportunities to receive compassion. Avoidance was associated with less likelihood of reporting opportunities to give and receive compassion and less positive responses to opportunities to give compassion. Those high in anxiety but simultaneously low in avoidance reported fewer self-compassionate actions, but we identified no further differences in compassionate action.

**Discussion:**

This study highlights the potential role of awareness of compassionate opportunities in attachment-related differences in compassion.

## Introduction

Caregiving is a central component of human nature, representing a fundamental element of intimate relationships (Weiss, 1980). One particularly important type of caregiving is compassion. While definitions of compassion are varied, with different components being emphasized by different researchers and traditions (see Strauss et al., 2016 for an overview), compassion has often been defined as sensitivity to suffering in the self or others with a motivation to act to alleviate that suffering (Gilbert, 2014). The ability to give and receive compassion is fundamental to sustaining life (Gilbert, 2005; Goetz et al., 2010) and critical for thriving mental and physical health and wellbeing (Cosley et al., 2010; MacBeth and Gumley, 2012). According to Bowlby (1988), the capacity to develop meaningful emotional connections with others as a caregiver and care-seeker is a critical prerequisite for mental wellbeing. Importantly, a critical determinant of this capacity is believed to be a healthy attachment style (Bowlby, 1969; Gillath et al., 2005; Mikulincer and Shaver, 2017).

Attachment styles are emotional and behavioral patterns in close relationships with profound effects on psychological and social wellbeing (Bowlby, 1969; Hazan and Shaver, 1987) and internal working models of the self and others (see Bartholomew and Horowitz, 1991; Mikulincer, 1995). Differences in attachment in adults can be characterized in relation to two orthogonal dimensions: anxiety and avoidance, which are characterized by fear of rejection and discomfort with closeness, respectively. Those with low anxiety and avoidance are described as having a secure attachment characterized by comfort with closeness and confidence in consistent availability of support from others. In contrast, those high in either anxiety, avoidance, or both are described as insecure. Both anxious and avoidant insecure attachments are characterized by doubt in the availability of consistent emotional support from close others.

In the last 50 years, researchers have identified significant associations between attachments and the capacity to give compassion to others and oneself (for reviews, see Mikulincer and Shaver, 2003, 2007, 2017; Lathren et al., 2021; Varley et al., 2024). In relation to giving compassion to others, laboratory-based research shows that those with anxious attachments often provide compassion and caregiving less effectively than secure individuals, often engaging in compulsive caregiving – an overbearing and intrusive means of offering care and compassion (Kunce and Shaver, 1994; see Mikulincer and Shaver, 2017 for a review). This compulsive caregiving is believed to be driven by a tendency to be overly focused on one’s own personal distress and attachment needs for proximity and security (Collins and Read, 1994; Shaver and Mikulincer, 2002; Mikulincer and Shaver, 2017). Overall, most research suggests that anxious individuals are aware of needs for compassion in others and often endorse altruistic motivations for compassion (Feeney and Collins, 2003; Feeney et al., 2013), but provide compassion less effectively than secure individuals. However, there is also evidence suggesting that anxious individuals may be less responsive to needs for compassion in others. Specifically, Feeney and Collins (2001) identified that anxiously attached individuals were less likely to show support to a partner in need, especially when the partner’s support seeking is less obvious, and were less likely to provide effective emotional support. These findings show similarities to patterns observed in avoidantly attached individuals.

Studies suggest those with avoidant attachments provide compassion less often than securely attached individuals (Mikulincer and Shaver, 2017). Relative to more secure individuals, those high in avoidance report being less likely to maintain proximity and provide support to a partner in need and are often less sensitive to the needs of a partner (Simpson et al., 1992; Kunce and Shaver, 1994; Feeney and Collins, 2001; Millings et al., 2013; Péloquin et al., 2014). These tendencies are believed to be driven by negative beliefs about the availability and responsiveness of others, discomfort with closeness, and adaptive suppression of attachment needs and negative emotions (Shaver and Mikulincer, 2002; Mikulincer and Shaver, 2019). Overall, research suggests avoidant individuals provide compassion to others less often, and that anxious individuals provide compassion less effectively than those with secure attachments.

Insecurely attached individuals also report less self-compassion than secure individuals (see Lathren et al., 2021 for a review). Multiple studies have linked both anxiety and avoidance to lower self-compassion (e.g., Raque-Bogdan et al., 2011; Wei et al., 2011; Pepping et al., 2015; Joeng et al., 2017a,b; Homan, 2018; Brophy et al., 2020). Relatedly, those who experienced abuse in childhood or who recall early caregivers lacking warmth or showing signs of indifference or rejection also report lower self-compassion (Tanaka et al., 2011; Potter et al., 2014; Pepping et al., 2015; Westphal et al., 2016).

Links between attachment insecurity and difficulty receiving compassion from others have been less widely studied, but some research suggests a relationship. For example, research indicates those with insecure attachments often doubt the availability and supportiveness of others (Collins and Read, 1990; Collins, 1996; Kelly et al., 2012). A recent meta-analysis also identified that attachment insecurity is positively associated with self-reported fear and discomfort related to receiving compassion from others (Varley et al., 2024). Further, studies have identified that those with insecure attachments show physiological threat responses to imagery tasks involving imagining receiving compassion from others (Rockliff et al., 2008; Baldwin et al., 2020) and that those with low recalled parental warmth report discomfort receiving compassion (Kelly and Dupasquier, 2016). Together, these studies suggest a likely link between attachment insecurity and difficulty receiving compassion from others.

Overall, previous studies implicate attachment insecurity in difficulties with compassion. However, several questions remain unaddressed. First, while the deleterious effects of attachment insecurity on self-compassion and giving compassion to others have been more widely studied, experiences related to receiving compassion from others have received little attention. This means that while research indicates that insecurely attached individuals feel discomfort or threat when receiving compassion, it remains unknown if they are less aware of opportunities to receive compassion and less likely to allow others to act compassionately toward them. This is also problematic as receiving compassion from others is not necessarily highly correlated with self-compassion and giving compassion to others. Indeed, these types of compassion have been acknowledged as distinct constructs (e.g., Steindl et al., 2021). Accordingly, previously identified relationships between attachment and self-compassion or giving compassion may not necessarily correspond with relationships between attachment and receiving compassion.

It is critical we understand not just attachment-related differences in capacities for self-compassion and giving compassion to others, but also the capacity to receive compassion. This is because attachment insecurity’s negative impact on wellbeing may be partially driven by inability to derive soothing from others. Evidence illustrates that mental wellbeing and abilities to regulate emotions and respond to threat are partially reliant on the quantity and quality of interactions with others (Cacioppo and Patrick, 2008; Cozolino, 2014; Siegel, 2020). Bowlby (1988) also argued that the capacity to develop emotional connections with others as both a caregiver and care-seeker is critical for mental wellbeing. Given that insecurely attached individuals feel discomfort related to receiving compassion (Varley et al., 2024), it is plausible these fears may impact awareness of opportunities to receive compassion or reduce the extent to which one allows others to act compassionately toward them. Clarifying this would provide useful information for the development of targeted clinical interventions mitigating negative impacts of attachment insecurity. This may be of particular utility, as while attachment styles are believed to develop in childhood and remain predominantly stable across life (Fraley et al., 2011), capacities for compassion can continue to be cultivated across the lifespan with benefits for wellbeing (Kirby, 2017).

Second, it is unknown how attachment insecurity relates to the experience of compassion in everyday life. Research to date has relied heavily on traditional retrospective-report and laboratory-based observational methods. Given that laboratory settings significantly differ from everyday life settings, it is unclear if previous inferences hold true in everyday life. It is important to clarify this as traditional retrospective-report and laboratory-based methods have limited ecological validity. In other words, the generalizability of such findings to the real world may be limited (Orne, 1962; Bronfenbrenner, 1977). Observing participants in laboratory settings inherently introduces bias as it is impossible for experimenters to entirely reproduce complex natural situations and because laboratory-based experiments are vulnerable to demand characteristics (Orne, 1962). Traditional retrospective-report methods are also susceptible to recall bias. The passing of time between an event of interest such as compassionate action in everyday life and completing a survey related to that event leaves these methods vulnerable to inaccuracy introduced by the fallibility of memory (Kahneman et al., 1982; Klein et al., 1992, 1996, 1997; Marsh and Yeung, 1998; Robinson and Clore, 2002).

Third, it is unknown how attachment may relate to awareness of opportunities for compassion (where ‘opportunities for compassion’ refers to awareness of suffering in the self or others and recognition that there is a chance to act to alleviate that suffering). So far, research has focused on examining attachment-related differences in compassionate actions. Unfortunately, this only captures one aspect of compassion, which has two major components: first, awareness of compassionate opportunities by being sensitive to suffering in oneself and others, and second, a commitment to alleviate and prevent that suffering through action (Gilbert, 2014, p. 19; see also Collins et al., 2006). Research has predominantly focused on how attachment impacts the second of these components. Consequently, it is unclear whether there are attachment-related differences in awareness of compassionate opportunities in addition to differences in compassionate action.

Understanding how attachment relates to awareness of compassionate opportunities rather than just compassionate actions would improve our understanding of the interrelation between attachment and the caregiving behavioral system and provide insight into how attachment influences the processing of information related to compassion (see Shaver and Mikulincer, 2002; Dykas and Cassidy, 2011 for models of attachment-related differences in the processing of information). This would have meaningful implications for clinical intervention. Understanding attachment-related differences in sensitivity to compassionate opportunities would provide greater insight into the stage/s of processing or the component of compassion with which attachment insecurity interferes – awareness of suffering/compassionate opportunities, or engagement in compassionate action. This could inform development and application of clinical interventions. For example, if avoidant individuals are less aware of compassionate opportunities, clinicians may be able to use this information to improve awareness of compassionate opportunities, facilitating greater engagement in compassionate action.

Overall, there is a clear need for ecologically valid research that reviews how attachment relates to the experience of compassion in everyday life, with a focus on both compassionate awareness and action across all three types of compassion: self-compassion, giving compassion to others, and receiving compassion from others. Consequently, our study aimed to investigate whether there are attachment-related differences in compassion-related experiences in everyday life settings, including the likelihood of reporting opportunities to be self-compassionate, to give compassion to others, and to receive compassion from others; in the likelihood of reporting acting self-compassionately, acting compassionately toward others, and allowing others to act compassionately toward oneself; and in the positivity of participants’ emotional responses to these compassionate opportunities.

To address our aim, participants completed a self-report measure of attachment (as attachment is a stable construct, making it less susceptible to issues associated with traditional retrospective-reports; Fraley et al., 2011) and a one-week experience sampling protocol in which participants were asked to complete five short experience sampling surveys each day for seven days. Experience sampling methods (ESM) achieve greater ecological validity than traditional retrospective-reports by asking participants to repeatedly report on everyday behavior and experiences close to the time of occurrence while going about their daily lives (Larson and Csikszentmihalyi, 1983; Csikszentmihalyi and Larson, 1987).

Each experience sampling survey asked participants whether they had observed opportunities to be compassionate to others, self-compassionate, and whether they had the opportunity to receive compassion from others. If participants reported observing compassionate opportunities, they were asked whether they engaged in compassionate actions toward others, toward themselves, or whether others had acted compassionately toward them. Participants also reported the perceived positivity of their emotional experience related to any compassionate opportunities.

We hypothesized that there would be a significant relationship between attachment and propensity to report opportunities for compassion. Given that avoidant individuals fear compassion (Varley et al., 2024) and adaptively suppress negative affective experiences (Shaver and Mikulincer, 2002; Mikulincer and Shaver, 2019), we predicted that greater attachment avoidance would be associated with less likelihood of reporting compassionate opportunities. Given the hypervigilance of anxiously attached individuals to possible signs of threat (Bowlby, 1973; Mikulincer and Shaver, 2003; Gillath et al., 2005; McWilliams and Brodeur, 2016), we predicted that attachment anxiety would be associated with greater likelihood of reporting compassionate opportunities.

We also anticipated a relationship between attachment and engagement in compassionate actions. As above, we predicted that attachment avoidance would have a negative association with the likelihood of engaging in compassionate actions. In contrast, given the tendency for those with anxious attachments to engage in compulsive caregiving, we anticipated that anxiety would have a positive association with the likelihood of engaging in compassionate actions.

Finally, given the dearth of previous research reviewing compassionate behavior in those high in both attachment anxiety and avoidance (sometimes referred to as fearful-avoidant attachment; Bartholomew and Horowitz, 1991), we used exploratory analyses to investigate whether there were any interactions between anxiety, avoidance and likelihood of reporting compassionate opportunities and actions. We also used exploratory analyses to investigate possible attachment-related differences in the positivity of participants’ emotional responses to any compassionate opportunities reported, including whether there were any interaction effects of attachment anxiety and avoidance on their emotional experiences. These analyses were unplanned, and we therefore did not have formal hypotheses. However, previous research utilizing traditional retrospective-report measures indicates that insecure attachment is associated with fear, aversion, and discomfort with compassionate experiences (Varley et al., 2024). It would therefore be plausible that the same pattern might appear in everyday life.

## Method

This study was pre-registered and received ethical approval from an institutional review board. Preregistration details, analysis code, and materials are linked here: https://osf.io/tm4cx/?view_only=a7be53dbb2e54a72a5c13d1ba8aeb8fb.

### Design

We used a mixed design with an observational cross-sectional design to measure attachment and a signal-contingent intensive longitudinal design to measure compassion in everyday life.

### Participants

We recruited 136 Australian university students via an undergraduate psychology research participation program. To be eligible, participants were required to: (1) have no prior experience with meditation, (2) be at least 18 years of age, (3) be fluent in English (as study materials were in English), and (4) have access to a smartphone they were willing to use to participate in the study. Participants received course credit for participation. Eleven participants could not complete the study as their phone was incompatible with the application used for ESM surveys. There was no further attrition.

Our final sample included 125 participants (95 women, 27 men, 3 non-binary, *M*_age_ = 18.74, *SD*_age_ = 1.66). Self-reported racial distribution included 67 participants who identified as White, 47 as Asian, five as mixed-race, one as Black, one as Pacific Islander, and four belonging to other racial backgrounds. Regarding education, 107 participants reported holding a high school diploma, 11 a Bachelor degree, three non-University tertiary qualifications, three less than a high school level of education, and one did not disclose.

Sample size selection was guided by an *a priori* power analysis for logistic multilevel models with two Level-2 (between-person) continuous predictors (attachment anxiety and avoidance). We ran 1,000 Monte Carlo simulations in Olvera Astivia et al.’s (2019) web application with parameter estimates guided by Arend and Schäfer’s (2019) recommendations. Simulations indicated that a minimum sample of 125 participants with at least 17 observations per participant would be required to identify medium effect sizes (*d* = 0.30) with 80% power at a significance criterion of α = 0.05.

### Procedure

Participants attended an initial laboratory session in which they received information about the study, provided consent, and completed a demographics questionnaire and measure of attachment. Participants also received training for completing ESM surveys using the phone application SEMA3 (Koval et al., 2019). Participants were informed they would receive five ESM surveys per day for one week, starting the following day, that each survey would take approximately two minutes, and they would have 30 min to complete each survey when prompted. Participants were informed they would receive a notification to their phone when a survey was available for completion, and if not completed after 15 min they would receive a second notification. Participants were asked to complete as many surveys as possible and were informed they would receive more course credit if they spent more time completing surveys to encourage higher compliance.

Starting the next day, participants completed seven consecutive days of ESM surveys. The SEMA3 application sent participants notifications to complete a survey five times daily using a pseudo-random signal-contingent schedule, with surveys scheduled at stratified pseudo-random intervals between 9:00 a.m. and 9:00 p.m. These hours were split into five equal intervals, with one survey sent in each interval. We programmed SEMA3 to not send two surveys within 30 min of each other, but surveys were otherwise sent at random times in each interval. To ensure clarity for participants, each survey included written definitions of the terms ‘compassion’ and ‘suffering’ prior to presenting the survey questions. These definitions were as follows:

In this study, we define ‘compassion’ as: *Sensitivity to suffering in the self or others, with a commitment to try and alleviate or prevent it*. We define ‘suffering’ as any kind of distress, discomfort, hardship, or pain. Suffering can be very mild (e.g., feeling slightly sad), very severe (e.g., breaking a bone, losing a close family member), or anywhere in between.

### Measures

#### Attachment

To assess attachment, participants completed the Experiences in Close Relationships Questionnaire-Revised (ECR-R; Fraley et al., 2000). The scale includes 36 items (e.g., “I’m afraid that I will lose my partner’s love”) to which participants indicate agreement on a 7-point Likert scale (1 = *strongly disagree*, 7 = *strongly agree*). Responses to two 18-item subscales are averaged to provide two continuous scores: one for anxiety, one for avoidance. Both subscales have excellent internal consistency (α_anxiety_ = 0.95, α_avoidance_ = 0.93; Sibley and Liu, 2004). In this study, internal consistency was good (α_anxiety_ = 0.86) and excellent (α_avoidance_ = 0.93).

#### Experience sampling surveys

We used single-item scales because longer surveys reduce data quality in ESM studies (Eisele et al., 2022) and single-items are considered acceptable for ESM due to repeated sampling (Csikszentmihalyi and Larson, 1987). Each item was appraised for content validity by the last author, an expert in compassion research. We also used qualitative feedback from pilot testing to ensure items were clear and interpreted consistently. Each survey included the following.

##### Interactions

Participants reported how many people they interacted with in the 30 min prior to each survey (“How many people have you interacted with [including face-to-face, telephone, text message, or via social media, etc.] in the last 30 min?”). Participants were able to respond with a number between zero and five or indicate “More than 5.” If participants responded with zero, they were only asked the questions below relevant to self-compassion.

##### Compassionate opportunities

Participants were asked to report whether they had compassionate opportunities for each type of compassion, including opportunities for compassion for others (“In the past 30 min, did you have the opportunity to be compassionate towards somebody?”), opportunities to receive compassion (“In the past 30 min, did you have an opportunity to receive compassion from someone?”), and opportunities for self-compassion (“In the past 30 min, did you have an opportunity to be compassionate to yourself?”). For each item, participants responded on a binary scale (0 = *no*, 1 = *yes*). Each item was scored separately to create three binary outcome variables.

##### Compassionate actions

For each type of compassion, if participants reported having had a compassionate opportunity, they were asked if they acted compassionately toward others (“Did you do something to help the person suffering (i.e., take some kind of action)?”), if they allowed others to act compassionately toward them (“Did you allow that person to act compassionately towards you?”), and if they acted self-compassionately (“Did you act in a compassionate way towards yourself when this opportunity arose?”) Participants responded on a binary scale (0 = *no*, 1 = *yes*). Each item was scored separately to create three binary outcome variables.

##### Emotional experience

If participants reported compassionate opportunities, they were asked about the quality of the respective emotional experience related to any opportunities to be compassionate to others (“In relation to the opportunity to give compassion to others, was the emotional experience you felt positive or negative?”), to receive compassion from others (“In relation to the opportunity to receive compassion, was the emotional experience you felt positive or negative?”), and to be self-compassionate (“In relation to the opportunity to be compassionate to yourself, was the emotional experience you felt positive or negative?”) Participants responded on a 7-point Likert scale (1 = *extremely negative*, 7 = *extremely positive*). Each item was scored separately to create three continuous outcome variables.

### Data analysis

To account for nesting of survey responses within participants we used generalized linear mixed effect models with logit link functions for binary responses and linear mixed effect models for continuous responses using *lme4* (Bates et al., 2015), *lmerTest* (Kuznetsova et al., 2017), and *performance* (Lüdecke et al., 2021) in R (Version 4.2.2; R Core Team, 2022). We used separate models for each outcome variable (likelihood of reporting each type of compassionate opportunity, each type of compassionate action, and the positivity of participants’ emotional responses to each type of compassionate opportunity). All models included random intercepts for participants. Attachment anxiety, avoidance, and their interaction term were included as Level-2 (between-person) fixed effects. Anxiety and avoidance scores were grand-mean centred. Significant interactions were followed-up by comparing effects at meaningful levels of the interacting variable (-1*SD*, mean, and + 1*SD*). We also conducted supplementary analyses with gender entered as a Level-2 (between-person) fixed effect to check for any gender-related differences across our outcome variables, as some previous research has identified that women report less self-compassion than men (Neff and Vonk, 2009; Neff and McGehee, 2010), but may be more likely to report compassion for others than men (Eisenberg and Lennon, 1983).

## Results

### Descriptive information

Overall, participants completed 63.02% of the ESM surveys (2,757 out of 4,375 surveys, an average of 22 surveys per person). The number of compassionate opportunities and actions reported by participants is in Table 1. Means and standard deviations of variables are in Table 2. We identified no gender-related differences for any of our studied outcomes (see Supplementary material for results).

**Table 1:** Summary of the number of times participants reported compassionate opportunities and actions.

Variable	Yes (% of Total)	No (% of Total)	Total
Self-compassionate opportunities	398 (14.99)	2,257 (85.01)	2,757
Self-compassionate actions	289 (72.80)	108 (27.20)	397
Opportunities for compassion for others	522 (19.23)	2,193 (80.77)	2,715
Compassionate actions toward others	381 (73.27)	139 (26.73)	520
Opportunities for compassion from others	236 (8.82)	2,439 (91.18)	2,675
Compassionate actions from others	181 (77.02)	54 (22.98)	235

**Table 2:** Means and standard deviations for measured variables.

Variable	*M*	SD
Attachment anxiety	3.83	0.93
Attachment avoidance	3.06	1.00
Self-compassionate opportunities	0.18	0.39
Self-compassionate actions	0.96	0.19
Opportunities for compassion toward others	0.20	0.40
Compassionate actions toward others	0.95	0.22
Opportunities for compassion from others	0.12	0.32
Compassionate actions from others	0.98	0.14
Emotional experience (compassion toward others)	5.09	1.36
Emotional experience (compassion from others)	4.85	1.55
Emotional experience (self-compassion)	4.34	1.50

Compassionate opportunities and actions were measured as binary variables (0 = no, 1 = yes). Consequently, means for these variables reflect average probabilities of responding ‘yes’.

### Compassionate opportunities

There was a significant positive effect of attachment anxiety on the likelihood of reporting all three types of compassionate opportunities such that higher anxiety was associated with greater likelihood of reporting compassionate opportunities (see Table 3). There was no effect of avoidance on likelihood of reporting opportunities for self-compassion. However, there was a significant negative effect of avoidance on the likelihood of reporting opportunities to give compassion to others and receive compassion from others. There were no interactions between anxiety and avoidance.

**Table 3:** Summary of the effects of attachment anxiety, avoidance, and their interaction on the likelihood of reporting compassionate opportunities.

Compassionate opportunities	*β*	*SE*	95% CI	*β_exp_*	*z*	*p*
For self						
Intercept	−1.92	0.10	−2.12, −1.72	0.15	−18.85	<0.001***
Anxiety	0.38	0.10	0.17, 0.58	1.46	3.61	<0.001***
Avoidance	−0.16	0.10	−0.36, 0.04	0.85	−1.58	0.113
Anxiety × avoidance	−0.09	0.10	−0.30, 0.11	0.91	−0.90	0.369
For others						
Intercept	−1.53	0.08	−1.69, −1.37	0.22	−18.75	<0.001***
Anxiety	0.33	0.09	0.16, 0.50	1.39	3.87	<0.001***
Avoidance	−0.25	0.08	−0.41, −0.09	0.78	−3.02	0.003**
Anxiety × avoidance	−0.02	0.09	−0.19, 0.15	0.98	−0.25	0.804
From others						
Intercept	−2.58	0.12	−2.81, −2.34	0.08	−21.60	<0.001***
Anxiety	0.40	0.12	0.17, 0.63	1.49	3.38	<0.001***
Avoidance	−0.31	0.11	−0.54, −0.09	0.73	−2.75	0.006**
Anxiety × avoidance	0.00	0.12	−0.23, 0.22	1.00	−0.03	0.974

*p < 0.05, **p < 0.01, ***p < 0.001.ICC, adjusted intraclass correlation coefficient. ICC_self_ = 0.17, ICC_for others_ = 0.11, ICC_from others_ = 0.17.

### Compassionate actions

**Figure 1:**
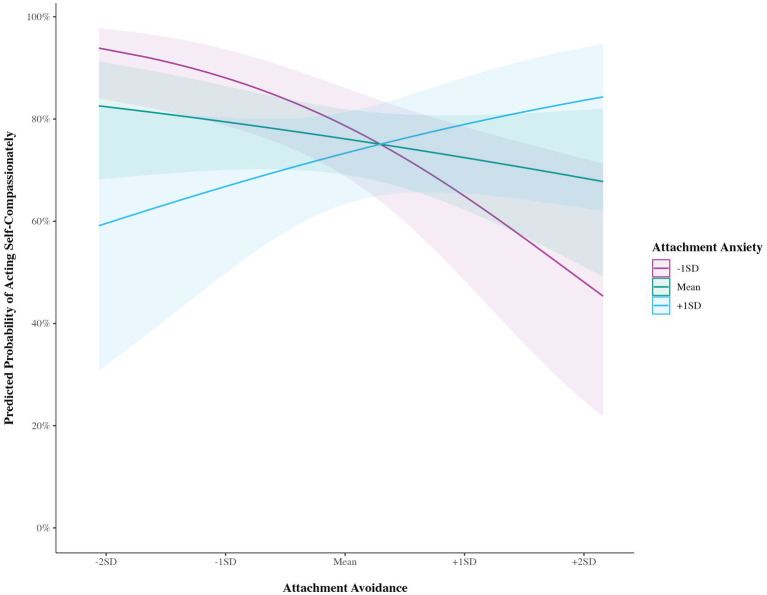
Effect of attachment anxiety on the predicted probability of acting self-compassionately at differing values of attachment avoidance.

There were no effects of attachment anxiety or avoidance on the likelihood of reporting any type of compassionate action (see Table 4). There were also no interactions between anxiety and avoidance on the likelihood of reporting acting compassionately toward others or allowing others to act compassionately toward oneself. However, there was a significant interaction between anxiety and avoidance on the likelihood of reporting self-compassionate actions. Simple slopes indicated that, at below average values of attachment avoidance, those with above average attachment anxiety were significantly less likely to act self-compassionately with an odds ratio of 0.50 (*β* = −0.70, *SE* = 0.27, 95% CI [−1.22, −0.18], *p* = 0.009; see Figure 1). However, the slope of anxiety on likelihood of acting self-compassionately did not differ significantly from zero at average (*β* = −0.16, *SE* = 0.19, 95% CI [−0.53, 0.21], *p* = 0.392) or above average avoidance (*β* = 0.38, *SE* = 0.27, 95% CI [−0.16, 0.92], *p* = 0.164).

**Table 4:** Summary of the effects of attachment anxiety and avoidance on likelihood of reporting compassionate actions.

Compassionate actions	*β*	*SE*	95% CI	*β_exp_*	*z*	*p*
For self						
Intercept	1.19	0.19	0.82, 1.57	3.30	6.30	<0.001***
Anxiety	−0.16	0.19	−0.53, 0.21	0.85	−0.86	0.392
Avoidance	−0.31	0.17	−0.64, 0.02	0.73	−1.84	0.066
Anxiety × avoidance	0.54	0.20	0.16, 0.92	1.72	2.77	0.006**
For others						
Intercept	1.20	0.15	0.90, 1.50	3.33	7.85	<0.001***
Anxiety	−0.21	0.15	−0.50, 0.09	0.81	−1.40	0.163
Avoidance	−0.22	0.14	−0.50, 0.06	0.80	−1.54	0.124
Anxiety × avoidance	−0.21	0.15	−0.49, 0.08	0.81	−1.40	0.161
From others						
Intercept	1.51	0.29	0.94, 2.08	4.53	5.20	<0.001***
Anxiety	−0.25	0.24	−0.72, 0.22	0.78	−1.06	0.291
Avoidance	−0.12	0.22	−0.54, 0.30	0.89	−0.55	0.584
Anxiety × avoidance	0.22	0.24	−0.24, 0.69	1.25	0.95	0.344

*p < 0.05, **p < 0.01, ***p < 0.001.ICC, adjusted intraclass correlation coefficient. ICC_self_ = 0.18, ICC_for others_ = 0.14, ICC_from others_ = 0.22.

### Emotional experience related to compassion

There was no significant effect of attachment anxiety on reported emotional experience in relation to self-compassion and giving compassion to others (see Table 5). However, there was a significant effect of attachment anxiety such that greater anxiety was associated less positive reported emotional experiences related to receiving compassion from others. There was no significant effect of attachment avoidance on the reported emotional experience related to self-compassion or receiving compassion from others. However, there was a significant effect of avoidance such that greater attachment avoidance was associated with less positive reported emotional experiences related to giving compassion to others. There were no interactions between anxiety and avoidance.

**Table 5:** Summary of the effects of attachment anxiety and avoidance on reported positivity of participants’ emotional experience in relation to opportunities for compassion.

Emotional experience	*β*	*SE*	95% CI	*t*	*p*
For self					
Intercept	4.43	0.11	4.20, 4.65	38.67	<0.001***
Anxiety	−0.10	0.12	−0.34, 0.14	−0.80	0.425
Avoidance	−0.13	0.11	−0.35, 0.09	−1.15	0.255
Anxiety × avoidance	0.06	0.12	−0.18, 0.29	0.48	0.634
For others					
Intercept	5.11	0.08	4.95, 5.27	62.49	<0.001***
Anxiety	0.00	0.09	−0.17, 0.17	−0.04	0.965
Avoidance	−0.24	0.08	−0.41, −0.08	−2.90	0.005**
Anxiety × avoidance	−0.09	0.09	−0.26, 0.08	−1.05	0.297
From others					
Intercept	4.92	0.12	4.67, 5.16	39.56	<0.001***
Anxiety	−0.27	0.13	−0.53, −0.01	−2.05	0.042*
Avoidance	−0.11	0.12	−0.35, 0.12	−0.94	0.349
Anxiety × avoidance	0.05	0.13	−0.20, 0.31	0.43	0.671

*p < 0.05, **p < 0.01, ***p < 0.001.ICC, adjusted intraclass correlation coefficient. ICC_self_ = 0.34, ICC_for others_ = 0.17, ICC_from others_ = 0.16.

## Discussion

We aimed to investigate attachment-related differences in the likelihood of reporting compassionate opportunities and actions in everyday life and in the positivity of emotional experiences related to compassionate opportunities in everyday life across all three types of compassion: self-compassion, giving compassion, and receiving compassion from others.

As anticipated, those higher in attachment anxiety were more likely to report opportunities for self-compassion, giving compassion, and receiving compassion. Additionally, as expected, those higher in avoidance were less likely to report opportunities to give and receive compassion, but surprisingly, avoidance had no association with reporting opportunities for self-compassion. Further, surprisingly, neither anxiety nor avoidance was associated with the likelihood of giving compassion or allowing others to act compassionately toward oneself. However, there was a significant interaction between anxiety and avoidance on the likelihood of reporting self-compassionate actions such that those with above-average anxiety but below-average avoidance had a lower likelihood of reporting acting self-compassionately. Additionally, attachment anxiety was associated with a less positive emotional experience related to opportunities to receive compassion from others and avoidance was associated with a less positive emotional experience related to opportunities to give compassion to others.

Our findings suggest that attachment anxiety may lead to greater awareness of needs for compassion in everyday life or greater sensitivity to suffering in the self and others. This aligns with research using traditional laboratory-based methods establishing that anxious individuals are more sensitive to emotional expressions than less anxious individuals (Fraley et al., 2006). These findings may result from anxious difficulties down-regulating distress or hypervigilance toward cues that are relevant to assessing and monitoring whether significant others are available and responsive as a means of attempting to maintain proximity to others (Shaver and Mikulincer, 2002; Mikulincer and Shaver, 2003, 2019; Fraley et al., 2006).

Relatedly, Varley et al. (2024) argued that anxious individuals may feel uncomfortable with compassionate experiences because they may interpret the need for compassion in the self or others as a possible threat to one’s ability to maintain proximity to others. Similarly, anxious individuals may be vigilant to cues indicating a need for compassion because these cues may be perceived as potentially signs of threat. This sense of threat may impair one’s ability to act compassionately. This may explain why highly anxious individuals are more likely to notice the need for compassion in themselves and opportunities to receive compassion from others but are not also more likely to act self-compassionately or to report allowing others to act compassionately toward them.

This interpretation may also explain why those high in anxiety but simultaneously low in avoidance are less likely to act self-compassionately. In the absence of the avoidant tendency to suppress negative affective experiences (Mikulincer and Shaver, 2019), the difficulty those high in anxiety have with down-regulating distress may result in prolonged hyperactivation of the attachment system which is thought to interfere with activation of the caregiving system (Gillath et al., 2005), which may then impair ability to act self-compassionately.

These results extend on previous research by more closely pinpointing how differences in attachment relate to differences in compassion-related behaviors. Specifically, our results imply that previous findings that anxiously attached individuals act compassionately less effectively than secure adults (Mikulincer and Shaver, 2017) may not be attributable to lack of awareness of suffering in oneself and others and that previous findings that anxious individuals sometimes engage in compulsive caregiving may be partially attributable to hypervigilance to suffering in others. These findings also support the theory that other issues such as experiencing personal distress in response to suffering may impede the effective provision of compassion (Monin et al., 2010; Vilchinsky et al., 2010), leading these individuals to be less equipped to act self-compassionately or to be soothed by the receipt of compassion from others.

Our research also supports the findings of previous research suggesting that those with greater attachment avoidance are less sensitive to the needs of others (e.g., Kunce and Shaver, 1994; Feeney and Collins, 2001; Braun et al., 2012; Millings et al., 2013; Péloquin et al., 2014), possibly due to discomfort engaging with others’ suffering (Mikulincer and Shaver, 2016; Varley et al., 2024). This may be due to avoidantly attached individuals lacking comfort with closeness and holding negative working models of others (Bartholomew and Horowitz, 1991; Brennan et al., 1998). Further, those with avoidant attachments suppress negative affective states that activate attachment needs (e.g., needs for closeness, support; Mikulincer and Shaver, 2016). This kind of suppression would make it difficult to bring awareness to the suffering of others and recognize opportunities for compassion. Interestingly, however, those with greater avoidance were not less likely to report opportunities for self-compassion. This suggests that while these individuals may suppress negative affective states (Mikulincer and Shaver, 2016), this may occur after having awareness of one’s own suffering. In contrast, our finding that avoidantly attached individuals were less likely to report opportunities to give compassion may suggest that previous findings indicating that avoidant individuals provide less compassion to others may be at least partially attributable to reduced awareness of the suffering of others, rather than an intentional decision to not act compassionately. Further, these individuals report fewer opportunities to receive compassion from others. This may indicate that outward expression of one’s suffering may be inhibited, limiting their ability to access opportunities to receive compassion from others. This aligns with Shaver and Mikulincer’s (2002) assertions and research (Mikulincer and Shaver, 2019) indicating that avoidant individuals use deactivating attachment strategies such as adaptive suppression of thoughts and feelings related to vulnerability or need because of beliefs in others being unavailable or unresponsive.

Overall, our findings in relation to attachment avoidance align with the assertions of Shaver et al. (2010) that if one’s caregiving system develops under unfavorable circumstances, they are likely to become less compassionate with respect to other people’s needs and suffering due to a lack of parental modeling, support, or positive social interactions. However, we argue that, at least in everyday life, this disruption to the development of the caregiving system may result in less awareness of the need for compassion, rather than more conscious choices to not act compassionately.

Our findings also meaningfully extend our understanding of attachment’s relationship with compassion by suggesting that, in everyday life, those with higher anxiety and avoidance are not less likely to act compassionately toward others if they are aware of the opportunity to do so. Indeed, previously identified attachment-related differences in compassionate action may have been attributable to previously unmeasured differences in awareness of compassionate opportunities or sensitivity to suffering. As noted, most previous research only assessed one component of compassion – compassionate action (or, in the case of retrospective-report measures, memories or beliefs about one’s compassionate motivations and actions). Our study extended on previous research by measuring both components of compassion – awareness of compassionate opportunities and compassionate action and by accounting for awareness of compassionate opportunities in our assessment of compassionate action. Thus, this is the first study to review attachment-related differences in compassionate action after accounting for awareness of compassionate opportunities. Consequently, it is possible that we generally observed no differences in compassionate action because attachment-related differences may occur at the level of awareness, rather than the level of action. For example, those with higher avoidance appear to be less sensitive to compassionate opportunities, and thus would be likely to act less compassionately overall.

Thus, our results provide the novel contribution of highlighting the type of compassion-related difficulties insecure individuals experience in everyday settings. Specifically, Gilbert (2014) has argued that effective compassion requires two qualities: first, sensitivity, involving being attuned to and able to accurately understand a person’s signals of need, and second, responsiveness, involving taking useful action, and validating another’s needs and feelings (or one’s own needs and feelings). Earlier researchers also emphasized these same two qualities as underpinning effective caregiving in general (Ainsworth et al., 1978; Reis and Shaver, 1988; Collins et al., 2006). Our results suggest those with insecure attachments likely experience difficulty predominantly with sensitivity.

### Limitations and suggestions for future research

While this study provides new information about attachment-related differences in compassion, its limitations should also be discussed. First, while asking participants to report on recent behaviors mitigates issues with traditional retrospective-report methods, ESM data is still subject to the ability of participants to have insight into and correctly recall their own actions and emotions. This prevents us from discerning whether participants sometimes report no compassionate opportunities because they were unaware of opportunities even when present rather than there genuinely being no opportunities. While we can infer that more avoidant individuals report fewer opportunities and more anxious individuals report more opportunities than more secure people overall, we cannot entirely exclude the possibility that this may reflect differences in the number of opportunities encountered. However, given that we gathered data at varying time points over an entire week, and we only gathered data related to opportunities to give and receive compassion when participants confirmed they had interacted with others, it seems less likely that this would be an artefact of avoidant individuals consistently encountering fewer and anxious individuals consistently encountering more opportunities than secure individuals. Nonetheless, it would be beneficial to explore methods that could mitigate both the ecological validity issues with laboratory-based methods and the lack of objective information when using ESM and traditional retrospective-reports. For example, *in situ* observational methods could clarify how often participants are unaware of opportunities for compassion when they are present and how contextual factors may influence awareness of suffering and engagement in compassionate actions.

Further, our data is correlational, and we are not able to make causal conclusions. It would be beneficial to investigate whether studies using experimental manipulations would identify a similar pattern of findings. For example, previous research has established that contextual activation of attachment security (i.e., ‘security priming’) can lead to greater responsiveness toward a romantic partner (Mikulincer et al., 2013, 2014). It would be pertinent to see if the same effects may emerge in everyday settings, and if they would vary across differences in attachment such that priming security may increase awareness of compassionate opportunities in those high in avoidance and reduce awareness in those high in anxiety. If so, this could provide a promising avenue for development of therapeutic interventions for mitigating negative outcomes associated with attachment insecurity and difficulties with compassion (e.g., more frequent arguments with partners, more negative affect in relationships, less relationship longevity; Levy and Davis, 1988; Kirkpatrick and Hazan, 1994; Simpson et al., 1996).

Relatedly, given we identified links between attachment insecurity and differences in awareness of compassionate opportunities, it may be beneficial to investigate whether relevant interventions may impact compassionate awareness. For example, application of compassionate mind training techniques from Compassion Focused Therapy may be useful as these aim to foster awareness of compassionate opportunities, engagement in compassionate actions, and a compassionate mindset toward the self and others while also targeting relevant inhibitors of effective compassion such as feeling distress in response to suffering (Gilbert, 2009). Clinicians may also benefit from using attachment-informed formulations to select appropriate techniques. For example, a clinician working with an anxious individual may choose to utilize habituation or cognitive restructuring techniques to lessen hypervigilance toward possible suffering, manage feelings of distress, or re-interpret ambiguous cues more positively. Conversely, a clinician working with an avoidant individual may utilize mindful awareness techniques to bring greater awareness to the needs of themselves and others.

It would also be beneficial for future research to investigate potential attachment-related differences in compassionate motivations in everyday life. While our study provided new information about attachment-related differences in the propensity to report compassionate opportunities and actions, it remains unclear if attachment-related differences in compassionate motivations emerge in everyday life. It also remains unclear whether any differences in motivation may moderate relationships between propensity to report compassionate opportunities and actions in everyday life, or whether differences in motivation may make some individuals more or less likely to notice compassionate opportunities.

The generalizability of our findings should also be considered as the sample recruited for this study were undergraduate students who were predominantly young women. Given this, our findings may differ in middle or late adulthood, in non-students or those with different levels of education, or in men. Future research should review whether associations between attachment and compassionate opportunities and actions vary in other samples. This would be valuable as research remains equivocal as to whether factors like gender, age, and education history lead to systematic differences in compassion-related outcomes. For example, while we identified no gender-related differences, other researchers have identified that women reported greater compassion for others than men (e.g., Pommier et al., 2019) but lower self-compassion than men when these constructs are measured with traditional self-reports (e.g., Neff and Vonk, 2009; Neff and McGehee, 2010). Further, while research suggests that capacities for compassion are present from childhood and early adolescence (see Roeser et al., 2018 for a review) and attachment styles are mostly stable across adolescence into adulthood (Fraley et al., 2011; Jones et al., 2018; Waters et al., 2021), other research has identified that older adults report higher levels of compassion for others than younger adults (Reiter et al., 2017). Considering this ambiguity, our results can be said to provide meaningful information about associations between attachment and compassionate opportunities and actions for young women in emerging adulthood. It is also possible that the same patterns of results may emerge for other populations, but further work is required to confirm this.

Finally, it ought to be acknowledged that a scale that provides separate measures of positive and negative affect may have been a more appropriate measure to assess emotional experiences in relation to compassionate opportunities. While our use of a single-item scale with a bidirectional response scale aimed to reduce participant burden, studies of affective structure have identified two orthogonal dimensions of affect – positive affect and negative affect (Watson and Tellegen, 1985; Watson et al., 1988). While our study was able to provide broad information about emotional experiences in relation to compassionate opportunities, in future, use of measures that measure both positive and negative affect (such as the Positive and Negative Affect Scales; Watson et al., 1988) may be able to provide more nuanced information and new insight into potential attachment-related differences in both positive and negative affective responses to compassionate experiences.

## Conclusion

This was the first study to investigate relationships between attachment and both components of compassion across all three forms of compassion in everyday life. We identified that greater attachment anxiety is associated with greater likelihood of reporting compassionate opportunities across all three forms of compassion while greater avoidance is associated with less. However, the likelihood of engaging in compassionate action was generally not a function of individual differences in attachment, with only one minor exception, with those high in anxiety but low in avoidance being less likely to act self-compassionately.

## Data availability statement

The datasets presented in this study can be found in online repositories. The names of the repository/repositories and accession number(s) can be found in the article/Supplementary material.

## Ethics statement

The studies involving humans were approved by the University of Queensland Human Research Ethics Committee. The studies were conducted in accordance with the local legislation and institutional requirements. The participants provided their written informed consent to participate in this study.

## Author contributions

DV: Conceptualization, Data curation, Formal analysis, Investigation, Methodology, Project administration, Software, Visualization, Writing – original draft, Writing – review & editing. CS: Conceptualization, Methodology, Supervision, Writing – review & editing. JK: Conceptualization, Methodology, Supervision, Writing – review & editing.
