# Supplementary material for: Attachment and propensity for reporting compassionate opportunities and behavior in everyday life
Source: Front Psychol. 2024 Jul 31;15:1409537. doi: 10.3389/fpsyg.2024.1409537 (PMC11322129; doi:10.3389/fpsyg.2024.1409537)
Supplement: Supplementary file 2 [file Table_2.DOCX]

**Supplementary Document**

Preregistration details, R code used for analyses, and study materials are available via the Open Science Framework here: <https://osf.io/tm4cx/?view_only=a7be53dbb2e54a72a5c13d1ba8aeb8fb>.

Below we report the results of analyses checking for gender-related differences in our studied outcome variables.

**Compassionate Opportunities**

There was no significant effect of gender on the likelihood of reporting all three types of compassionate opportunities (see Table S1).

**Table S1**

*Summary of the effects of gender on the likelihood of reporting compassionate opportunities.*

| **Compassionate Opportunities** | ***β*** | ***SE*** | **95% CI** | ***β_exp_*** | ***z*** | ***p*** |
| --- | --- | --- | --- | --- | --- | --- |
| **For self** |  |  |  |  |  |  |
| Intercept (Women) | -1.96 | 0.12 | -2.19, -1.72 | 0.14 | -16.42 | <.001*** |
| Men | 0.17 | 0.25 | -0.31, 0.65 | 1.19 | 0.70 | .481 |
| Non-binary | 0.15 | 0.64 | -1.11, 1.41 | 1.17 | 0.24 | .811 |
| **For others** |  |  |  |  |  |  |
| Intercept (Women) | -1.49 | 0.10 | -1.68, -1.31 | 0.22 | -15.71 | <.001*** |
| Men | -0.17 | 0.21 | -0.57, 0.23 | 0.85 | -0.82 | .414 |
| Non-binary | 0.56 | 0.51 | -0.45, 1.57 | 1.76 | 1.10 | .274 |
| **From others** |  |  |  |  |  |  |
| Intercept (Women) | -2.51 | 0.14 | -2.77, -2.24 | 0.08 | -18.45 | <.001*** |
| Men | -0.22 | 0.28 | -0.77, 0.34 | 0.81 | -0.76 | .450 |
| Non-binary | -0.31 | 0.77 | -1.83, 1.20 | 0.73 | -0.40 | .686 |

*Note. ***p* < .001. *ICC* = adjusted intraclass correlation coefficient. *ICC*_self_  = 0.19, *ICC*_for others_  = 0.13, *ICC*_from others_  = 0.20.

**Compassionate Actions**

There was no significant effect of gender on the likelihood of reporting all three types of compassionate actions (see Table S2).

**Table S2**

*Summary of the effects of gender on the likelihood of reporting compassionate actions.*

| **Compassionate Actions** | ***β*** | ***SE*** | **95% CI** | ***β_exp_*** | ***z*** | ***p*** |
| --- | --- | --- | --- | --- | --- | --- |
| **For self** |  |  |  |  |  |  |
| Intercept (Women) | 1.31 | 0.23 | 0.87, 1.76 | 3.71 | 5.78 | <.001*** |
| Men | -0.11 | 0.42 | -0.94, 0.71 | 0.89 | -0.27 | .787 |
| Non-binary | -0.22 | 1.05 | -2.28, 1.84 | 0.80 | -0.21 | .835 |
| **For others** |  |  |  |  |  |  |
| Intercept (Women) | 1.17 | 0.16 | 0.86, 1.48 | 3.23 | 7.43 | <.001*** |
| Men | -0.37 | 0.31 | -0.98, 0.25 | 0.69 | -1.16 | .245 |
| Non-binary | 1.91 | 1.16 | -0.36, 4.18 | 6.77 | 1.65 | .099 |
| **From others** |  |  |  |  |  |  |
| Intercept (Women) | 1.63 | 0.30 | 1.00, 2.22 | 5.09 | 5.39 | <.001*** |
| Men | -0.71 | 0.49 | -1.70, 0.25 | 0.49 | -1.46 | .145 |
| Non-binary | -0.27 | 1.45 | -3.10, 2.57 | 0.76 | -0.19 | .852 |

*Note. ***p* < .001. *ICC* = adjusted intraclass correlation coefficient. *ICC*_self_  = 0.27, *ICC*_for others_  = 0.14, *ICC*_from others_  = 0.20.

**Emotional Experience**

There was no significant effect of gender on reported emotional experience in relation to any of the three types of compassion (see Table S3).

**Table S3**

*Summary of the effects of gender on reported positivity of participants’ emotional experience in relation to opportunities for compassion.*

| **Emotional Experience** | ***β*** | ***SE*** | **95% CI** | ***t*** | ***p*** |
| --- | --- | --- | --- | --- | --- |
| **For self** |  |  |  |  |  |
| Intercept (Women) | 4.39 | 0.13 | 4.14, 4.65 | 34.03 | <.001*** |
| Men | 0.18 | 0.27 | -0.34, 0.70 | 0.67 | .503 |
| Non-binary | -0.20 | 0.68 | -1.54, 1.14 | -0.30 | .769 |
| **For others** |  |  |  |  |  |
| Intercept (Women) | 5.12 | 0.09 | 4.94, 5.30 | 55.07 | <.001*** |
| Men | -0.05 | 0.20 | -0.45, 0.35 | -0.24 | .813 |
| Non-binary | 0.08 | 0.48 | -0.86, 1.01 | 0.16 | .873 |
| **From others** |  |  |  |  |  |
| Intercept (Women) | 4.96 | 0.13 | 4.70, 5.22 | 37.15 | <.001*** |
| Men | -0.33 | 0.30 | -0.92, 0.25 | -1.12 | .267 |
| Non-binary | 0.04 | 0.86 | -1.65, 1.73 | 0.05 | .963 |

*Note. ***p* < .001. *ICC* = adjusted intraclass correlation coefficient. *ICC*_self_  = 0.35, *ICC*_for others_  = 0.19, *ICC*_from others_  = 0.16.
